# Factors Affecting Definitions of and Approaches to Integrative Medicine: A Mixed Methods Study Examining China's Integrative Medicine Development

**DOI:** 10.1155/2015/458765

**Published:** 2015-02-22

**Authors:** Weijun Zhang, Sonya E. Pritzker, Ka-Kit Hui

**Affiliations:** Department of Medicine, Center for East-West Medicine, University of California, Los Angeles, CA 90024, USA

## Abstract

*Aim*. This study identifies existing definitions and approaches among China's integrative medicine (IM) experts and examines relationships with key characteristics distinguishing individual experts. *Methods*. Snowball sampling was used to select 73 IM experts for semistructured interviews. In this mixed methods study, we first identified definitions and approaches through analyzing core statements. Four key factors, including age, education, practice type, and working environment, were then chosen to evaluate the associations with the definitions. *Results*. Four unique definitions were identified, including IM as a “new medicine” (D1), as a combination of western medicine (WM) and Chinese medicine (CM) (D2), as a modernization of CM (D3), and as a westernization of CM (D4). D4 was mostly supported by those working in WM organizations, while D3 was more prominent from individuals working in CM organizations (*P* = 0.00004). More than 64% clinicians had D2 while only 1 (5.9%) nonclinician had D2. Only 1 clinician (1.8%) had D4 while almost 30% nonclinicians had D4 (*P* = 0.0001). Among nonclinicians working in WM organizations, 83.3% of them had D4 (*P* = 0.001). *Conclusion*. Findings indicate that institutional structure and practice type are factors affecting IM approaches. These results carry implications for the ways in which western countries move forward with the definition and implementation of IM.

## 1. Introduction

Defining integrative medicine (IM) has always been a challenge to the field. Over many years, definitions have ranged from care that “combines treatments from conventional medicine and [complementary/alternative medicine] CAM for which there is some high-quality evidence of safety and effectiveness” [1] to care that is fundamentally person-centered and holistic in nature and which utilizes “all appropriate therapeutic approaches” [2]. Likewise, while some definitions emphasize the importance of scientific evidence in IM, others emphasize the integration of ancient metaphysical medical principles, regardless of scientific evidence [3]. For some, IM exists as a paradigm shift, a restructuring on par with the creation of a new medicine. Others are more skeptical, distrusting the move to construct scientific evidence based on ancient principles [4]. For yet others, IM can be understood as a movement back to the roots of medicine, as it “focuses on preventive maintenance of health by paying attention to all relative components of lifestyle… it insists on patients being active participants in their healthcare” [5]. Finally, there has been a more recent trend to talk about “integrative healthcare” rather than integrative medicine since the current shift to population health demands a broader term [6].

There is also a lack of consensus about the appropriate structures and processes of implementing IM. Some argue for a model of IM that offers patients an array of alternatives in a single clinic or multiple linked clinics, while others maintain that simply combining different modalities in the care of patients, alone, “is not integrated medicine” [7]. Most IM practices adopt a team-oriented approach, with a focus on facilitating collaboration between conventional medicine practitioners and CAM practitioners [8–10]. Dual-trained physicians, including MDs with training in a complementary practice or field, have been shown to be more oriented to creating collaborative working environments for IM [11, 12]. Although there has been increased opportunity for dual-training, made possible especially by several recent educational grants funded by the National Center for Complementary and Integrative Health (NCCIH) as well as efforts in many of the medical and nursing schools that are part of Academic Consortium for Integrative Medicine and Health (ACIMH) [13–15], dual-trained physicians are scarce in the contemporary USA. There is, further, very little research on definitions and approaches of dual-trained physicians.

A major part of the problem with finding consensus on definitions of IM in the USA is the relative novelty of formal approaches. This is not the case in China, where IM has been meticulously planned and implemented by government in various institutional contexts over the last 60 years. As one of four integrative healthcare systems recognized by the World Health Organization (WHO) [16], China's IM practitioners and researchers are all dual-trained in both western biomedical medicine and Chinese medicine. In this mixed methods' study, we endeavored to identify various definitions of and approaches to IM that exist among IM clinical and research experts in China. Our results indicate that it is institutional structure and the demands of clinical practice as well as the opportunities for research funding and collaboration therein that play the largest role in the way IM is defined in the everyday worlds of Chinese practitioners. This finding has both practical and policy implications for the ways in which western countries move forward with the definition and implementation of IM.

## 2. Background: Integrative Medicine in China

In this section, we briefly review the structural development of “integrative Chinese and western medicine” (中西医结合—*zhongxiyi jiehe*) or IM in China since the 1950s, when the Chinese government first intervened to create official, institutionalized IM throughout the country. When the Communists came to power in 1949, uniting Chinese and western medical practitioners was planned as one of three major health policies. Efforts were first made to organize and establish control over the more than 450,000 traditional medical practitioners in China. Such practitioners were mostly in rural areas and, like those in urban areas, usually worked in a private practice setting. From 1950 to 1954, the government therefore emphasized teaching the rudiments of modern medicine and public health to traditional practitioners, bringing them into “united clinics/hospitals” where they officially worked alongside western biomedical practitioners. Many of these united clinics later became full-fledged CM or IM hospitals [17].

Starting in 1955, the central government encouraged western biomedical physicians to “broaden their knowledge” in traditional healing practices. This sparked the development of a systematic two-and-half year full-time training program called “western physicians learning Chinese medicine” in Beijing and other cities [18]. By 1962, 379 specially selected young doctors had graduated from the program, while more than 4,000 physicians from various specialties participated in an affiliated part-time program [19]. This group of biomedical physicians, together with the early group of traditional practitioners working at western biomedical hospitals, thus forming the group of IM pioneers.

After completing their training, these pioneers were assigned to different working environments, including both western biomedical (WM) and Chinese medical (CM) organizations, to further develop IM. In WM settings, they worked at either newly established institutes of integrative medicine or departments of Chinese medicine within WM research institutes, hospitals, and universities. At the time, CM organizations were all newly established and included hospitals, CM colleges, and research institutions such as the China Academy of Traditional Chinese Medicine (now China Academy of Chinese Medical Sciences, CACMS), established in 1955. Within these settings, many dual-trained physicians practiced IM, while others focused exclusively on research and teaching [19, 20]. Since that time, IM units within WM organizations and CM organizations expanded very quickly. Accordingly, in 1986 the State Administration of Traditional Chinese Medicine (SATCM), which regulates all clinical services in CM organizations and provides guiding principles on education and research related to CM and IM for all institutions, was established independent of the Ministry of Health [21]. Currently, the number of CM colleges and universities in China has increased to 35, including one in each province. Likewise, 46 medical universities have IM and CM programs, housed within special departments or institutes. In addition, there are 358 integrative medicine hospitals and 3015 CM hospitals operating in China [22], and among the roughly 15,000 western biomedical hospitals 95% of them include departments of CM. At present, around 40% of the therapies prescribed in WM hospitals are traditional medicine. Similarly, in CM hospitals, around 40% of all prescribed drugs are biomedical [23].

In terms of training, CM colleges currently offer five-year courses for undergraduates, with roughly 30–40% of time devoted to teaching biomedicine, modern scientific principles, and experimental methodologies [24]. In 2013, there were 1133 graduates with Ph.D. degrees, 10389 graduates with three-year master degrees, and 84585 undergraduates with bachelor degrees in CM [22]. Likewise, of the 123 colleges of western medicine in contemporary China, about 10–15% of curricular time is devoted to CM [23]. Between 1956 and the present, then, multiple IM educational programs in CM and WM organizations have produced IM physicians and researchers with vastly differing ranges of training and experience within applied integrative settings. In 2013, there were 168 graduates with master degrees in IM and 38 graduates with Ph.D. degrees in IM from WM organizations [22]. The IM workforce in China thus includes two major dual-trained groups with different educational backgrounds. The first includes practitioners originally trained in biomedicine with additional training in CM. The second includes practitioners originally trained in CM with additional training in biomedicine. As more IM programs are instituted in CM organizations, the latter are dominating the IM workforce in contemporary China. There are also a small number of experts with nonmedical training who conduct research in IM.

## 3. Methods

### 3.1. Study Design

The present study utilizes an exploratory sequential mixed methods design, wherein we first collected and analyzed qualitative data and then used these findings to inform subsequent quantitative analysis [25, 26]. The qualitative data consists of semistructured oral history interviews with Chinese IM pioneers and current leaders, while the quantitative analysis consists of a detailed statistical review of several key themes identified within interviewee responses. The semistructured interview guide utilized in these interviews consists of several open-ended questions with probes for clarification and additional details [27]. The interview questions inquired about both personal events and opinions related to integrative medicine in China. Five domains of inquiry were included: (1) biographic questions, including questions about family, educational background, and working experience; (2) IM medical and/or clinical training; (3) specialty insights into the integration of Chinese medicine and biomedicine; (4) opinions regarding and observations of the current development of integrative medicine in China; and (5) opinion on the future development of integrative medicine in China. The definition and description of approaches in integrative medicine fall into the third domain. The UCLA Institutional Review Board approved this study.

### 3.2. Sample

We used a snowball sampling methodology to develop two lists of participants for interviews [28]. Per our request, the Chinese Association of Integrative Medicine (CAIM) provided background materials on over 200 IM experts, including pioneers and current leaders. Based on this list, we created a spreadsheet summarizing key characteristics, including age, education, working environment, and key contributions in IM, for each expert. We then reviewed their publications to supplement and confirm our understanding of their contributions. Using their contributions and importance in the field as selection criteria, we first identified 34 first-generation experts, aged 70–90 who actively participated in the development of IM during late 1950s and early 1960s. With help from a six-member group specifically organized by the CAIM to facilitate communication with potential interviewees, all first-generation experts agreed to be interviewed. Using a snowball sampling methodology, we then consulted with these pioneers to generate a list of 42 current academic leaders who worked closely with pioneers in the past and are still active in the field of IM. In total, we collected 76 interviews. Three interviews were excluded from the study due to the fact that the interviewees' experience in IM totaled less than 10 years (two current leaders) or because of communication difficulties (one pioneer).

### 3.3. Data Collection

The majority of interviews with pioneers and current leaders were conducted in Mandarin Chinese, while some were conducted in English or a mixture of Chinese and English. Interviews were videotaped and recorded in audio format. The transcripts for the current research were based on recorded audio files; however researchers consulted video files for tone and expression as needed. Interviews varied in length, ranging from 30 minutes to three hours.

Interview questions were sent to interviewees well in advance of the scheduled interview for review, suggestions, and corrections. Based on the expertise of the particular interviewee, questions were tailored accordingly. Interviews opened with biographic questions. In each domain, interviewers began with broad topics and then probed each response, continuing to narrow questions until the information exhausted all responses or the topic changed.

### 3.4. Data Analysis

In this mixed methods study, we utilized a “quasi-statistical approach” to qualitative coding where themes are divided into categories and then statistically examines in association with key characteristics of interviewees [29]. This approach constitutes the integration of qualitative and quantitative data through data transformation [25].

#### 3.4.1. Qualitative Coding

All interviews were transcribed by independent transcriptionists. A research assistant audited all transcripts in Chinese against the original audiotapes. Another research assistant audited all transcripts in English against the original audiotapes. The interviewees subsequently had the opportunity to review the edited transcript to ensure factual accuracy and, if desired, to provide additional commentary. After receiving the corrected transcripts, a research assistant created tables of contents and indices for each transcript.

In our analysis, we used an exploratory technique in which transcripts were reviewed to identify a “core statement” illustrating the key concepts in each oral author's perspective on integrative medicine development in China. This exploratory technique, developed by Lincoln and Guba, involves grouping similar ideas or items into “piles” and then identifying and labeling the overarching themes or domains of each of the piles [30].

Based on content indexed by our research assistant, we extracted answers to the definition of integrative medicine. All raw materials were input into a spreadsheet and sorted into themes. Through this process, we identified four major definitions, which reflect the diversity of opinions expressed by the various types of experts. Based on the extracted answers, two investigators (Weijun Zhang and Ka-Kit Hui) independently coded the definition and approach to the interviewee, with an initial intercoder agreement kappa of 0.816 [31].

#### 3.4.2. Quantitative Analysis

Even with the kappa value of 0.816, we further analyzed the code disagreement to reach full agreement between the two coders. The agreed-upon codes (D1, D2, D3, and D4) in the master coding dataset were then used as dependent variables for the subsequent analysis. Previous research has indicated that age, education, and working environment are factors affecting providers' perception towards IM [11]; and clinicians and researchers may have different attitudes regarding IM [32]. We then chose four key characteristics (age, original education background, type of expert (clinician or nonclinician), and working environment) as independent variables to examine the association with definitions of and approaches to IM.

Statistical analyses were performed using statistical software, SAS version 9.3 (Cary, NC). The associations between the definition of IM espoused by each participant and each factor (age, original education background, type of experts, and working environment) were first evaluated individually using Fisher exact test. The effect of interactions between selected pairs of factors on the definition of IM was further investigated by examining the stratified associations in the corresponding subsets using Fisher exact tests. *P* values less than 0.05 were considered as statistically significant.

## 4. Results

### 4.1. Characteristics of Interviewees

Interviewees had a mean age of 67.3 years. The median age was 71 years old. 84.9 percent were male, while 15.1 percent were female. 64.4 percent of interviewees were originally trained in biomedicine, while 32.9 percent were originally trained in CM and 2.7 percent were trained in nonmedical fields. 45.2 percent of interviewees could be considered pioneers who developed and practiced IM in late 1950s and early 1960s. The other 54.8 percent could be considered current leaders (nonpioneers). 76.7 percent of interviewees were clinicians, while 23.3 percent were nonclinicians involved exclusively in research and education. 67.1 percent of interviewees were currently practicing or had recently practiced IM in a CM organization and 32.9 percent were practicing or had practiced in a WM organization. The characteristics of study participants are shown in Table 1.

### 4.2. Interviewees' Definition of Integrative Medicine

In our analysis, we identified four unique ways of defining IM among our interviewees, listed in Table 2.

#### 4.2.1. D1 Definition One: The Creation of a New Medicine

The first definition used by participants emphasizes the creation of an entirely “new medicine” by blending the theories and practices of western medicine and CM. One interviewee, who was working at a CM university, uses a chemistry metaphor to describe his thinking on the subject:

To use a metaphor: the gases hydrogen and oxygen in combination generate water, and once it is water, it is no longer gas, but liquid. In this instance, the form has undergone a dramatic transformation. Hydrogen and oxygen are no longer distinguishable. They have become something new. But if, to use another example, you combine green beans with soybeans, it is a mixture but you can still clearly differentiate each kind of bean…. Most people think that the highest level of integrative medicine is the formation of a new medicine, rather than a mixture or compound.

However, almost every interviewee who espouses this view agrees that the new medicine has to go through multiple stages of development in order to actualize. One interviewee, who had been trained in western biomedicine and worked in a biomedical organization, explains:

There are three distinct phases in the process of integrating Chinese and Western medicine(s). First, the basic compatibility of the medicines needs to be recognized by both sides. Second, an allowance must be made for this compatibility. [Because] modern Western medicine pays attention to local pathology, and focuses on removing it or attacking it, and Chinese medicine strives to treat the whole, focusing on regulating the internal system. Chinese medicine thus can supplement modern Western medicine in exactly the ways it needs to be supplemented. But Chinese medicine lacks the kind of rapid treatment that is found in modern Western medicine. So this complementarity is deserving of theoretical research… Therefore, [third] there is hope for developing new understandings of diseases and further creating a new therapeutic system that blends the strengths of both Chinese and Western medicines.

#### 4.2.2. D2 Definition Two: Use of Chinese and Western Medicine Alongside One Another

The second definition expressed by interviewees does not mention the creation of a new medicine. Instead, the approach associated with this definition argues that integrative practitioners need to understand both Chinese and western medicines, including the strengths and weaknesses of both approaches as well as the way each can be explained with each other. The first goal here is to meet patients' need for healing, regardless of approach, as one clinician who espoused this definition argues:

I wish I could give my patients the best treatments for relieving their suffering. If an approach is effective, safe, and reasonable theoretically, I think I will use it regardless of whether it is western medications or [CM] herbs.

Another goal encompassed within this definition is for researchers and clinicians to translate or explain each medical system to the other for better understanding. One pioneer and clinician, who worked in a CM organization, said,

Chinese medicine and Western medicine are two very different medical systems…. we can consider the mechanism of so-called “calming the liver and subduing yang” in the treatment of hypertension within the context of Western medicine. We can combine the strengths, and explain it theoretically such that Westerners can easily understand what “calming the liver subduing yang” means… if you study the effects of “settling the liver and extinguishing wind” in the nervous system, in the endocrine system, or even the effect on angiotensin levels, then communication will be much easier.

#### 4.2.3. D3 Definition Three: Improving Chinese Medicine with Current Technology

The third definition emphasizes the view that clinical practice and research should primarily follow the theories of Chinese medicine. This approach utilizes current technology to study patients and treatments with the overarching goal of improving Chinese medicine. As one interviewee, who graduated from a CM university and is currently working at a CM organization, stated:

Regardless of whether you are developing Chinese medicine or western medicine, you have to deal with how to utilize modern technology to enhance, enrich, and to develop certain specialties. Especially for Chinese medicine, a traditional medicine with a few thousand years of history… it has its own methodology. Therefore, whatever you are developing, modernization of Chinese medicine or integrative medicine, I think you should not ignore the principles of Chinese medicine. You have to follow these principles, utilizing modern technology and absorbing some of the methods from modern sciences to enrich Chinese medicine.

#### 4.2.4. D4 Definition Four: Westernization of Chinese Medicine

The fourth definition is described as the westernization of Chinese medicine. The approach here is that clinicians and researchers approach one of many modalities and/or mechanisms of Chinese medicine from a biomedical perspective. As one interviewee, a neuroscientist and acupuncture researcher working at a leading medical University in China, said:

To be honest, I did not spend two years to learn Chinese medicine like my predecessors… so I only scratched the surface. With help from my teachers, I started a project that I had never tried before. I studied the involvement of the hypothalamus area with acupuncture analgesia in rabbits, first using electrophysiological methods, and then using neuropharmacological methods. By combining these two methods, I investigated the effects of acupuncture needling on electrical activity in the hypothalamus, and how pain is reduced when two competing signals- pain and needling- are produced together.

Overall, all interviewees were more likely to define integrative medicine as D2 (pragmatic approach, 50.7%) and less likely to define it as D4 (westernization of CM, 8.2%).

### 4.3. Factors Relating to Different Definitions

#### 4.3.1. Differences between Pioneers and Current Leaders (Nonpioneers) as well as Age Groups

Overall, there were no significant differences between the definitions espoused by pioneers and current leaders; age alone was not associated with definition. However, no subject under 50 years expressed D1 (the creation of a new medicine).

#### 4.3.2. Differences between Practice Types

**Figure 1:**
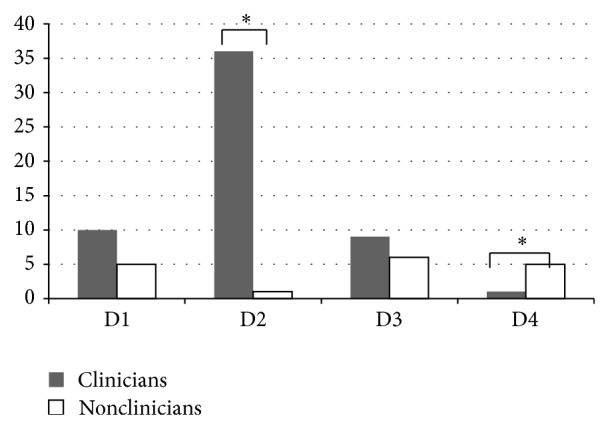
The definition differences between clinician and nonclinician.  ^*^Clinicians expressed more D2 than nonclinicians significantly; nonclinicians espoused more D4 than clinicians significantly. *P* = 0.0001.

Figure 1 provides the differences between clinicians and nonclinicians groups. More than 64% of clinicians expressed D2 (pragmatic combination) while only one (5.9%) nonclinician expressed this definition. On the other hand, only one clinician (1.8%) expressed D4 (westernization of CM) while almost 29.4% nonclinicians espoused this definition (*P* = 0.0001).

#### 4.3.3. Differences Based on Educational Backgrounds

Those interviewees with an initial education in western medicine were less likely to define IM as D3 (improving CM with current technology) than those with an initial education in CM. Only those with initial education in biomedicine expressed D4 (westernization of CM). However, there was no significant difference between groups when defining IM (Fisher exact test *P* = 0.072).

#### 4.3.4. Differences in Working Environments

**Figure 2:**
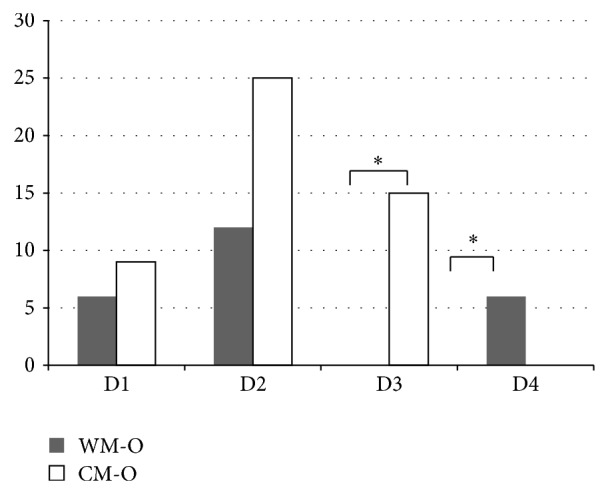
The definition differences between WM and CM working environment. WM-O: western medicine organizations. CM-O: Chinese medicine organizations.  ^*^D4 was only chosen by those working in WM-O; D3 was only chosen by those working in CM-O. *P* = 0.00004.

Figure 2 details the differences in interviewees' definitions of IM based on working environments: WM organizations or CM organizations. As might be expected, D4 was only chosen by those working in WM organizations, while D3 was only chosen by those working in CM organizations (Fisher exact test *P* = 0.00004).

### 4.4. Differences in Interactions of Factors

#### 4.4.1. Education Background and Practice Type

**Figure 3:**
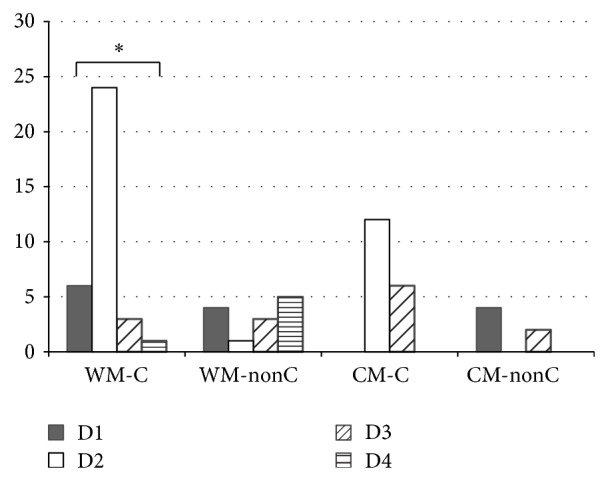
The definition differences between subgroups in education background and practice type. WM-C: clinician whose original education was in western medicine. WM-nonC: nonclinicians whose original education was in western medicine. CM-C: clinician whose original education was in Chinese medicine. CM-nonC: nonclinicians whose original education was in Chinese medicine.  ^*^WM-C expressed more D2 than other definitions significantly. *P* = 0.0001.

Among those with an initial education in biomedicine, more than 70% of clinicians expressed D2 (pragmatic combination), while nonclinicians were roughly evenly distributed among the other three definitions and were a little more likely to have D4 (westernization of CM), followed by D1 (new medicine) and D3 (improving CM with current technology) (Fisher exact test *P* = 0.0001), as shown in Figure 3. For nonclinicians with an initial education in CM, two out of six nonclinicians expressed D3 and another four expressed D1. No one expressed D4 and D2 (Fisher exact test *P* = 0.12).

#### 4.4.2. Working Environment and Practice Type

**Figure 4:**
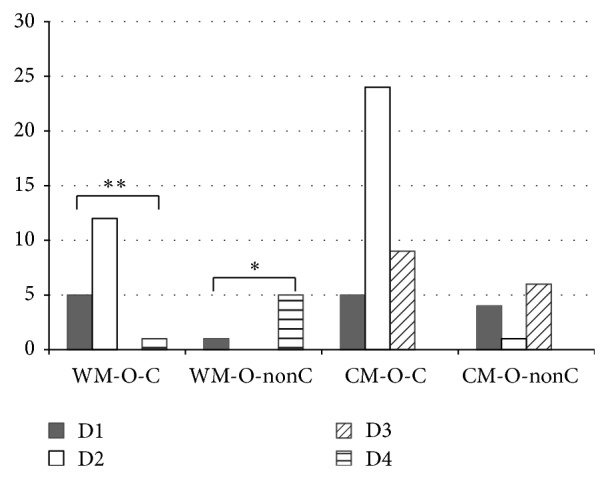
The definition differences between subgroups in practice types and working environment. WM-O-C: clinicians working in WM organizations. WM-O-nonC: nonclinicians working in WM organizations. CM-O-C: clinicians working in CM organizations. CM-O-nonC: nonclinicians working in CM organizations.  ^*^WM-O-nonC expressed D4 significantly more than other definition. *P* = 0.001.  ^**^WM-O-C expressed D2 significantly more than other definitions. *P* = 0.030.

Almost all nonclinicians working in WM organizations expressed D4 (westernization of CM), excluding one who expressed D1 (new medicine), while those working in CM organizations were about equally split between D1 and D3 (Fisher exact test *P* = 0.001). Among clinicians working in WM organizations, most expressed D2 (pragmatic combination), followed by D1, while one expressed D4. Clinicians working in CM organizations also commonly expressed D2 but more favored D3 (Fisher exact test *P* = 0.030). Details are shown in Figure 4.

We also tried to analyze the two-way interactions of practice types, original education background, and working environment. There was no nonclinician with an educational background in CM who worked in a WM organization; only two nonclinicians had been educated originally in Chinese medicine and worked in CM organizations; and only two clinicians who had been educated originally in Chinese medicine worked in western medicine organizations.

## 5. Discussion

### 5.1. Understanding the Definitions: A Historical Perspective

The term “integrated Chinese and western medicine” (*zhongxiyi jiehe*) was first mentioned in Chinese literature in 1959 [33]. Originally, this term emphasized the creation of an entirely “new medicine” by blending the theories and practices of biomedicine and CM, a concept corresponding to the first definition found in the current study. Since, at the time, the notion was emanating from top political leaders, many in the field took great cultural pride in creating the new medicine [34]. Idealism is clear in this definition, however. As this ideal was put into practice, awareness quickly emerged that the creation of a new medicine could not be immediate. In 1999, a theory of “three phases” was thus developed, in which only the third phase reaches the development of a “new medicine,” while the first two phases include a slow integration in attitudes, knowledge, skills, and theory [35]. Definitions two, three, and four thus emerged over time as individual practitioners began to apply IM in various settings.

There are key differences between definitions two, three, and four, however. The second definition represents a pragmatic approach to IM that is less concerned with ideal goals and more focused on immediate application in a clinical context. It provides guidance to practitioners, such that they know when, where, what, and how to use each medicine appropriately [36]. On one hand, this approach is an ongoing integration drawing upon both medicine types in hope to gradually optimize treatments in clinical practice. In many ways, it can be regarded as the second phase of the three phases in the first definition [35]. On the other hand, this approach, as an important aspect of integration, also uses modern scientific methods to assist traditional diagnoses and to interpret clinical mechanisms of action. In the pragmatic approach to IM, concepts are translated—often in practice—between the two medicines, making it more possible for practitioners of each medicine to understand the terminologies and practices of the other [37].

The third definition also takes a pragmatic stance on the development of IM, prioritizing classical CM theories and clinical methodologies at the same time as recognizing the value of approaching CM with modern technology in order to improve upon it. In the third definition, however, there is a clear prioritization of classical and traditional terminology, theory, and practice. CM is made better with biomedical technology, in this approach, to the extent that its core features remain undisturbed [38, 39]. What exactly “undisturbed” means, however, can be controversial and depends greatly upon different practitioners' individual experience and perspective.

In the fourth definition—the westernization of CM—we see a different type of pragmatic approach to understanding how CM and biomedicine relate to one another. This approach emphasizes the study of CM methods vis-à-vis their effects on the biomedical body. The terminology, theory, and practice used here are overwhelmingly biomedical. In contrast to the third definition, the values and knowledge of CM here are subordinated to those of biomedicine [40]. This definition is similar to that of NCCIH in the USA, where CAM practice with high-quality evidence of safety and effectiveness can be absorbed into conventional medicine [1].

The differentiation between the third and fourth definitions may seem subtle, given that both use methodologies in western medicine and modern science to assist in studying Chinese medicine [41]. This is an issue that has been controversial and hotly debated in China. For some extremists, integrating CM with biomedical technologies will result in the loss of the essence of CM. This loss, in their view, will lead to the eventual abandonment of traditional medicine, and they therefore consider any form of integration to be a westernization of CM [42]. For others, biomedical approaches to research and practice are simply tools to further develop CM. And, for still others, the development of CM along biomedical lines is a key to the formation of a viable IM [41]. Moreover, while the actions and publications of specific researchers are often inspired by a certain definition and approach, the ways in which they are interpreted by other practitioners and researchers are often controversial. For example, the analysis of* artemisinin* (derived from wormwood) resulted in the demonstration of the antimalarial effects of a western drug in 1971. Even though the inventor herself (also an interviewee) thought her approach exemplified D3—or the modernization of CM with contemporary technology [43]—many others interpreted her work as westernization of CM. This demonstrates the complexity of what IM means to specific individuals, especially after they have had many years of clinical and research experience that blur the lines of original ideals.

### 5.2. Key Factors Affecting Definitions and Approaches

#### 5.2.1. Clinical Practice

From our results, it is clear that, in clinical environments where practitioners are required to think on their feet in the care of patients, the definition of IM quickly moves from an idealized vision of a “new medicine” into a pragmatic approach that uses CM and biomedicine alongside one another in whatever way most benefits the patient. This is also inspired, to a large extent, by the rising demands of patients for combined approaches to care [44]. IM clinicians are thus forced in everyday interactions to cultivate a dual focus, often dubbed “double diagnosis and double treatment.” The double diagnosis in IM, which means including a disease diagnosis in biomedicine and pattern diagnosis in CM for each patient, was formalized in 1973 by one of our interviewees [36]. This approach allows individual doctors to strategize how to construct “integrative” treatment targeted at multiple aspects of illness wherein CM and biomedicine can be used in a complementary fashion. As Karchmer has observed, clinicians are required to note the double diagnosis and double treatment plans in the patients' medical records [45]. It is not surprising, therefore, that the second definition was most often supported by clinicians.

#### 5.2.2. Research Practice

The present results suggest that practical implementation of IM research in China demands a distinct approach to the definition of IM that prioritizes either western biomedicine or CM. Unlike clinicians, researchers have to seek research funds from various related government agencies. Overarching government funding priorities thus inevitably have impact on how nonclinician researchers understand IM. From this perspective, the government's support for analyzing effective properties of various CM treatments using modern scientific concepts and techniques, along with the adoption of international research standards for randomized controlled trials (RCT), has clearly had an impact on the ways in which nonclinician researchers define IM. Depending on where their funding sources come from, researchers are more apt to choose definitions three or four.

#### 5.2.3. Structures and Institutions

Structural limitations and institutional set-up play an important large, if not sometimes larger, role in how IM is implemented in China or elsewhere. Our results suggest that working environment is a key factor influencing the definitions and approaches of individual practitioners of IM. This relates to health policy and regulation at a systems level, working environment at an organizational level, and interaction with peers at an individual level.

As one of four integrative healthcare systems recognized by the WHO [16], health policy and regulatory frameworks in China seek to balance western biomedicine and Chinese medicine in the development of specific health systems. “Equal emphasis on Chinese medicine and western medicine” and “promoting integrative medicine” are two goals consistently supported at the highest levels of leadership [19, 46]. Modernization, however, is another important Chinese health policy that encourages WM organizations to participate and requires that CM organizations work towards it [47].

Besides widespread national health policies, other guidelines and plans deriving from the SATCM also influence how clinical practice and research are conducted in both CM and CM-primary IM organizations. For example, due to the privatization of the healthcare market, utilization of CM decreased by over 40% in the 1990s, especially in urban areas [48]. In response to this challenge, the SATCM put a great deal of effort into protecting and expanding the roles of CM practitioners in the healthcare system, including the institution of the requirement that 70% of clinical services provided by CM hospitals should be CM, and 50% of services provided by IM hospitals should be CM; 70% of clinical staff in CM hospitals are required to be trained in CM; 60% of clinical staff in IM hospitals should be trained in CM or IM [49, 50]. Likewise, in order to promote the modernization of CM in both research and practice, the Ministry of Science and Technology (MOST) joined the Ministry of Health and the SATCM, along with several other ministries, to draft the Outline of Traditional Chinese Medicine Innovation and Development Plan (2006–2020). The outline guides innovation, modernization, and globalization efforts at the national level [51].

The balancing of high level government demands for integration and modernization can prove challenging within specific institutions, especially given the overall sentiment that overt westernization of CM is politically incorrect [42]. IM practitioners at CM organizations are thus required to maintain an uneasy balance between two contradictory mandates: to represent the rich legacy of Chinese culture and to meet the modern demands of scientific sophistication [52]. Based on the huge impact of modernization of CM at systems and organizational level as well as the “bad reputation” of the westernization of CM, it is easy to understand that the fourth definition was not chosen by those in CM organizations.

IM units at WM organizations are usually staffed by graduate students that were trained in their department or institutes [53]. Compared to IM at CM organizations, IM at WM organizations is thus unique in a few key ways. First, clinical practice, education, and research at these institutions are regulated and guided by both the Ministry of Health and the SATCM. Second, there is no requirement for a certain percentage of CM services, simply a suggested distribution of services. Third, there are more funding sources for research grants compared to those who are in CM organizations, especially for basic researchers. Most importantly, for both IM clinicians and researchers, the majority of their peers within WM organizations are oriented towards biomedical principles. Their work thus naturally takes on a more biomedical character. Given the lack of widespread, open support for the westernization of CM, however, these individuals may hesitate to speak openly about their approach. However, in practice, they actually adopt the fourth definition. This is especially true for basic researchers, who are competing for funding and publications alongside biomedical researchers.

#### 5.2.4. Other Factors

The results did not support the hypothesis that age and pioneer status comprised a significant factor in individuals' definitions of IM. This runs contrary to expectations that pioneers may espouse a more idealistic definition of IM reflecting their training in the 1950s and 1960s. However, this result also suggests the importance of such pioneers' experience in framing their understanding of IM. Given their repeated frustrations in instantly developing a new medicine [46], these pioneers were therefore more apt to adopt, along with practitioners younger than 50 years old, a pragmatic approach to defining IM.

Surprisingly, our result suggested that educational background of dual-trained IM experts is not a significant factor influencing the definition of IM. This differs from the results obtained in a Korean study, which demonstrated that educational background determines the way integration is understood and put into practice [54]. In China, educational background, compared to institutional set-up and healthcare system factors, may not be as significant.

### 5.3. Implications

The present study carries several implications. In the USA, where we are currently wrangling with the issue of how to apply IM in real clinical settings, the present study suggests that we have a great deal to learn from Chinese clinicians who have been dealing with this very issue for more than 60 years. Though policy and practice in the west will certainly be different than those in China, many of the pitfalls and advantages to the ways in which certain policies have been actualized in practice in China can provide a guiding light to thinking through the way we implement IM in the west.

Specifically, as the development of integrative approaches to care is being supported by organizations such as the WHO [55], the National Institutes of Health, and ACIMH [56], it is useful to look at the ways in which China's national policies on integration have influenced the clinical and research practices of individual clinicians over time. As we have shown here, for example, clinicians who are practicing IM are often thrown into environments where they are forced to make pragmatic decisions that strain even the best-intentioned integrative definitions. Influenced by policies dictating how much biomedicine must be practiced in a certain setting as well as by patient demands, what often happens in these clinical situations is that the whole-systems approach to Chinese medicine is often sacrificed. Learning to* do* IM in busy settings, in other words, is often much more difficult than imagined and relates especially to institutional structures. It would therefore behoove westerners seeking to implement IM in hospitals or clinics to undertake a more detailed study of Chinese institutions as well as clinicians and their experiences. Within such a study, we would learn a great deal about the benefits to diagnosing patients from both a biomedical and a traditional perspective, including especially the impact of identifying multiple pathophysiological subsets of disease on the success of care. We would likewise learn about the challenges such “double diagnosis, double treatment” approaches to care entail, including what is sometimes criticized as merely “lip service” to traditional medicine in the context of policies dictating certain integrative demands on physicians from above. The present study thus provides one entry point into a more detailed appreciation of Chinese clinical experience.

In research, our results demonstrate that researchers examined in this study supported D4 (the westernization of Chinese medicine) when working in WM organizations, either because funding sources demanded such an approach or because they were influenced by the institutions in which they found themselves working. Similar demands and pressures exist for IM researchers in the west. The classic RCT was naturally adopted in clinical trials of Chinese medicine. However, researchers who are working in CM organization supported D3 (modernization of Chinese medicine). From our perspective, the modernization of Chinese medicine requires research designs to represent the principles of Chinese medicine, including whole systems and pattern differentiation (pattern diagnosis). The development of innovative approaches, such as whole systems research (WSR), offer promising opportunities for research to appropriately capture and measure the complexities of care in IM and other forms of CAM [57, 58]. However, subgroup analysis of pattern differentiation in clinical trials in western literature is rare even though it is popular in China. Whether a sound methodology can be developed to tailor pattern differentiation in clinical trial design remains to be seen. Likewise, research that attends to whether traditional or biomedical approaches to Chinese medicine or other forms of CAM are critical for developing the type of research that will best support the true integration of multiple medical systems in the west [59]. From our perspective, it is thus imperative that such efforts to develop innovative research approaches in IM be supported at both national and institutional levels.

Finally, our study also directs us towards the awareness that it would also be helpful, in trying to avoid some of the pitfalls that have made the practice of IM in China so fraught with controversy, to involve leaders other than MD specialists in the establishment of IM systems. Generalists as well as CAM specialists should thus also play a significant role in the development of policies and practices shaping IM's development in the west [56]. Our results likewise indicate that collaborative meetings and discussions involving both western and Chinese experts in the development of IM would allow both parties to learn as much as possible from each other and to create the best IM healthcare possible in specific local contexts. In sum, our results suggest that a balanced approach to the development IM organizations is essential, as is attention to the links between institutions. This also applies to the integration of conventional medicine with any form of CAM, such as Ayurveda, homeopathy, or anthroposophic medicine.

### 5.4. Limitations

Limitations of the present study include the fact that the snowball sampling for identifying interviewees yielded a small sample and skewed distribution, which prevented formal statistical tests of interaction effects. A larger caveat consists of the difficulties involved with attributing one exclusive definition to each participant. Many of the IM definitions shared in the course of our interviews move between the ideal and the practical in the sense of a desired future versus the realities of everyday practice in different settings. This truth is notwithstanding; we argue that the delineation of these definitions in the Chinese context holds value in that it allows us to distinguish the overarching approaches used to combine CM and biomedicine in China. From a conceptual standpoint, this delineation invites a dialogue regarding how various definitions do indeed impact the research and the practice of IM in any country.

## 6. Conclusion

The present study provides a glimpse into the thinking of dual-trained IM experts in a WHO recognized integrative healthcare system. The value of such perspectives does not necessarily lie in its demonstration of some unchanging truth claim that IM holds in China. Instead, because these ideas come from individuals with dual-training and a great deal of experience designing and implementing IM research and practice, this study offers the opportunity to think through the meaning of some of the different directions that IM research and clinical practice may lead in the United States and other western countries. In the USA, researchers and clinicians are well aware of the fact that idealized notions of creating a “new medicine” with IM are often compromised in various ways in the design of specific research studies and clinical systems. While idealized definitions are often the starting point, practical applications of IM are complex processes that develop over time and must be applied by individuals with adequate training in multiple domains. The suitable definition is likely to emerge and settle only as the practice of IM develops.

## Figures and Tables

**Table 1:** Characteristics of the interview sample (*n* = 73).

	Number of experts	Percent
Age, mean = 67.3, std. dev. = 13.7		
<50 years	11	15.1
50–65 years	21	28.8
>65 years	41	56.1
Gender		
Male	62	84.9
Female	11	15.1
Type of practice		
Pioneers	33	45.2
Colleagues and leaders	40	54.8
Original education		
Western medicine	47	64.4
Chinese medicine	24	32.9
Nonmedicine	2	2.7
Type of expert		
Clinicians	56	76.7
Nonclinicians	17	23.3
Working environment		
Western medicine	24	32.9
Chinese medicine	49	67.1

**Table 2:** The four definitions used by participants in the present study.

	Approach	Goal	a.k.a.
D1	Fully understand both western medicine and Chinese medicine and then blend the best medicines clinically and theoretically	To create a new medicine	Ideal or classic

D2	Fully understand the strength and weakness of both western and Chinese medicine and then utilize the best parts of both, depending on evidence or not	To better serve their patients or enhance communication between both kinds of medicine	Pragmatic

D3	Under the theoretical framework of Chinese medicine, utilize current technology to study patients and treatment	To improve and modernize Chinese medicine	Modernization of Chinese medicine

D4	Based on their area of specialty in western medicine, study modalities and/or mechanisms of Chinese medicine in clinical practice and research	To enhance and expand the scope of understanding of studied specialty	Westernization of Chinese medicine

## References

[1] National Center for Complementary and Alternative Medicine (2004). *Expanding Horizons of Health Care: Strategic Plan 2005–2009*.

[2] Kreitzer M. J., Kligler B., Meeker W. C. (2009). *Health Professions Education and Integrative Health Care*.

[3] Caspi O., Morrell P. (2001). Integrated medicine: orthodox meets alternative. *British Medical Journal*.

[4] Dalen J. E. (1999). Is integrative medicine the medicine of the future? A debate between Arnold S. Relman, and Andrew Weil. *Archives of Internal Medicine*.

[5] Snyderman R., Weil A. T. (2002). Integrative medicine: bringing medicine back to its roots. *Archives of Internal Medicine*.

[6] Boon H., Verhoef M., O'Hara D., Findlay B., Majid N. (2004). Integrative healthcare: arriving at a working definition. *Alternative Therapies in Health and Medicine*.

[7] Caspi O. (2001). Integrated medicine: orthodox meets alternative. Bringing complementary and alternative medicine (CAM) into mainstream is not integration. *British Medical Journal*.

[8] Horrigam B., Lewis S., Abrams D., Pechura C. (2012). *Integrative Medicine in America*.

[9] Boon H., Verhoef M., O'Hara D., Findlay B. (2004). From parallel practice to integrative health care: a conceptual framework. *BMC Health Services Research*.

[10] Mulkins A. L., Eng J., Verhoef M. J. (2005). Working towards a model of integrative health care: critical elements for an effective team. *Complementary Therapies in Medicine*.

[11] Hsiao A.-F., Ryan G. W., Hays R. D., Coulter I. D., Andersen R. M., Wenger N. S. (2006). Variations in provider conceptions of integrative medicine. *Social Science & Medicine*.

[12] Ben-Arye E. (2010). The role of dual-trained conventional/complementary physicians as mediators of integration in primary care. *Evidence-Based Complementary and Alternative Medicine*.

[13] Kreitzer M. J., Sierpina V. S. (2008). NCCAM awards grants to CAM institutions to enhance research education. *Explore: The Journal of Science and Healing*.

[14] Nedrow A. R., Heitkemper M., Frenkel M., Mann D., Wayne P., Hughes E. (2007). Collaborations between allopathic and complementary and alternative medicine health professionals: four initiatives. *Academic Medicine*.

[15] Sullivan B. M., Furner S. E., Cramer G. D. (2014). Development of a student-mentored research program between a complementary and alternative medicine university and a traditional, research-intensive university. *Academic Medicine*.

[16] World Health Organization (2002). *WHO Traditional Medicine Strategy 2002–2005*.

[17] Croizier R. C. (1965). Traditional medicine in Communist China: science, Communism and cultural nationalism. *The China Quarterly*.

[18] Ministry of Health (1958). Exploring the great treasure of traditional Chinese medicine: report of class of Western medicine physicians learning Chinese medicine. *Journal of Traditonal Chinese Medicne*.

[19] Wang Z. R., Li J. W., Chen K. J. (2010). The establishment of integrative medicine specialty. *The History of China's Integrative Medicine*.

[20] Gao T., Shiwaku K., Fukushima T., Isobe A., Yamane Y. (1999). Medical education in China for the 21st century. *Medical Education*.

[21] Chen K. J., Xu H. (2003). The integration of traditional Chinese medicine and Western medicine. *European Review*.

[22] State Administration of Traditional Chinese Medicine (2013). *China Statistical Yearbook of Chinese Medicine*.

[23] Xu J., Yang Y. (2009). Traditional Chinese medicine in the Chinese health care system. *Health Policy*.

[24] Hesketh T., Zhu W. X. (1997). Health in China: traditional Chinese medicine: one country, two systems. *British Medical Journal*.

[25] Fetters M. D., Curry L. A., Creswell J. W. (2013). Achieving integration in mixed methods designs—principles and practices. *Health Services Research*.

[26] Creswell J. W., Clark V. L. P., Creswell J. W., Clark V. L. P. (2011). Choosing a mixed methods design. *Designing and Conducting Mixed Methods Research*.

[27] Bauman L. J., Adair E. G. (1992). The use of ethnographic interviewing to inform questionnaire construction. *Health Education Quarterly*.

[28] Biernacki P., Waldorf D. (1981). Snowball sampling: problems and techniques of chain referral sampling. *Sociological Methods & Research*.

[29] Miller W., Crabtree B. (1992). *Primary Care Research: A Multi Typology and Qualitative Road Map*.

[30] Lincoln Y. S., Guba E. G. (1985). *Naturalistic Inquiry*.

[31] Krippendorff K. H. (2012). *Content Analysis: An Introduction to Its Methodology*.

[32] Jobst K. A. (2009). Becoming and growing—what does integration mean?. *The Journal of Alternative and Complementary Medicine*.

[33] Chen S. K. (2000). Ten achievements of integrative medicine for 50 tears in China. *Zhongguo Zhong Xi Yi Jie He Za Zhi*.

[34] Croizier R. C. (1973). *Traditional Medicine in Modern China: Social, Political, and Cultural Aspects*.

[35] Wu X. Z. (1999). Integrative medicine in the 21st century. *Medecine & Philosophy*.

[36] Shen Z. Y. (1973). Preliminary discussion on integrative medicine in internal medicine. *Journal of Traditonal Chinese Medicne*.

[37] Cai J. F. (1988). Integration of traditional Chinese medicine with Western medicine—right or wrong?. *Social Science and Medicine*.

[38] Wang J. P., Dai Z. X., Zhang Z. H. (1980). On modernization of traditional Chinese medicine. *Shanghai Journal of Traditional Chinese Medicine*.

[39] Yan J. H. (1999). How far can modernization of Chinese medicine go?. *Medicine and Philosophy (Humanistic & Social Medicine Edition)*.

[40] Stumpf S. H., Shapiro S. J. (2006). Bilateral integrative medicine, obviously. *Evidence-Based Complementary and Alternative Medicine*.

[41] Xie Z.-F. (2005). On the methodology for integration of traditional Chinese and Western medicine. *Journal of Chinese Integrative Medicine*.

[42] Li Z. Z. (1998). Reconsideration on modernization of traditional Chinese medicine. *China Journal of Traditional Chinese Medicine and Pharmacy*.

[43] Tu Y. (2011). The discovery of artemisinin (qinghaosu) and gifts from Chinese medicine. *Nature Medicine*.

[44] Lu A.-P., Ding X.-R., Chen K.-J. (2008). Current situation and progress in integrative medicine in China. *Chinese Journal of Integrative Medicine*.

[45] Karchmer E. I. (2010). Chinese medicine in action: on the postcoloniality of medical practice in China. *Medical Anthropology: Cross Cultural Studies in Health and Illness*.

[46] Lu L. (1999). National health policy and organizational development in integrative medicine since new China. *China Historical Materials of Science and Technology*.

[47] Xu Q., Bauer R., Hendry B. M. (2013). The quest for modernisation of traditional Chinese medicine. *BMC Complementary and Alternative Medicine*.

[48] Jin L. (2010). From mainstream to marginal? Trends in the use of Chinese medicine in China from 1991 to 2004. *Social Science and Medicine*.

[49] State Administration of Traditional Chinese Medicine (2011). *Guideline for Integrative Medicine Hospitals*.

[50] State Administration of Traditional Chinese Medicine (2011). *Guideline for Developing Chinese Medicine in Western Medicine Hospitals*.

[51] Ministry of Science and Technology (2007). *Outline of Traditional Chiense Medicine Innovation and Development Plan (2006–2020)*.

[52] Quah S. R. (2003). Traditional healing systems and the ethos of science. *Social Science and Medicine*.

[53] Zhe G. Q. (2011). *Study on Factors Affecting on the Development of Department of Chinese Medicine at Western Medical Hospitals*.

[54] Lim J., Yun Y., Lee S., Cho Y., Chae H. (2013). Perspectives on medical services integration among conventional western, traditional Korean, and dual-licensed medical doctors in Korea. *Evidence-Based Complementary and Alternative Medicine*.

[55] World Health Organization (WHO) (2013). *WHO Traditional Medicine Strategy 2014–2023*.

[56] Anderson B., Given S., Jeffres A., Morris W., Goldstein M. S., Weeks J. (2013). Acupuncture and oriental medicine practitioners in primary care. *Meeting the Nation's Primary Care Needs: Current and Prospective Roles of Practitioners of Acupuncture and Oriental Medicine*.

[57] Pritzker S., Hui K. (2012). Building an evidence-base for tcm and integrative east-west medicine: a review of recent developments in innovative research design. *Journal of Traditional and Complementary Medicine*.

[58] Verhoef M. J., Lewith G., Ritenbaugh C., Boon H., Fleishman S., Leis A. (2005). Complementary and alternative medicine whole systems research: beyond identification of inadequacies of the RCT. *Complementary Therapies in Medicine*.

[59] Price S., Long A. F., Godfrey M., Thomas K. J. (2011). Getting inside acupuncture trials—exploring intervention theory and rationale. *BMC Complementary and Alternative Medicine*.

